# Computational Evaluation of Al-Decorated g-CN Nanostructures as High-Performance Hydrogen-Storage Media

**DOI:** 10.3390/nano12152580

**Published:** 2022-07-27

**Authors:** Peng Gao, Xihao Chen, Jiwen Li, Yue Wang, Ya Liao, Shichang Liao, Guangyu Zhu, Yuebin Tan, Fuqiang Zhai

**Affiliations:** 1 School of Materials Science and Engineering, Chongqing University of Arts and Sciences, Chongqing 402160, China; xihaochen@cqwu.edu.cn (X.C.); liao__ya@163.com (Y.L.); zhuguangyu@cqwu.edu.cn (G.Z.); 2 State Key Laboratory of Precision Spectroscopy, East China Normal University, Shanghai 200062, China; 3 School of Chemistry and Molecular Bioscience, University of Wollongong, Wollongong, NSW 2500, Australia; pg177@uowmail.edu.au; 4 Molecular Horizons, University of Wollongong, Wollongong, NSW 2500, Australia; 5 College of Physics and Electronic Engineering, Northwest Normal University, Lanzhou 730070, China; jiwenli369@163.com; 6 Department of Electrical Engineering, Hanyang University, Seoul 04763, Korea; wangyue9471@hanyang.ac.kr; 7 School of Materials and Energy, Southwest University, Chongqing 400715, China; lsc19980403@email.swu.edu.cn; 8 Department of Biochemistry and Molecular & Cellular Biology, Georgetown University, Washington, DC 20007, USA; ty157@georgetown.edu

**Keywords:** DFT, ab initio, molecular dynamics

## Abstract

Density functional theory (DFT) calculations were employed to solve the electronic structure of aluminum (Al)-doped g-CN and further to evaluate its performance in hydrogen storage. Within our configurations, each 2 × 2 supercell of this two-dimensional material can accommodate four Al atoms, and there exist chemical bonding and partial charge transfer between pyridinic nitrogen (N) and Al atoms. The doped Al atom loses electrons and tends to be electronically positive; moreover, a local electronic field can be formed around itself, inducing the adsorbed H_2_ molecules to be polarized. The polarized H_2_ molecules were found to be adsorbed by both the N and Al atoms, giving rise to the electrostatic attractions between the H_2_ molecules and the Al-doped g-CN surface. We found that each 2 × 2 supercell can adsorb at most, 24 H_2_ molecules, and the corresponding adsorption energies ranged from −0.11 to −0.31 eV. The highest hydrogen-storage capacity of the Al-doped g-CN can reach up to 6.15 wt%, surpassing the goal of 5.50 wt% proposed by the U.S. Department of Energy. Additionally, effective adsorption sites can be easily differentiated by the electronic potential distribution map of the optimized configurations. Such a composite material has been proven to possess a high potential for hydrogen storage, and we have good reasons to expect that in the future, more advanced materials can be developed based on this unit.

## 1. Introduction

Under the context of a global energy shortage and high emissions of greenhouse gases, finding high-performance sources of renewable energy has become increasingly crucial. Hydrogen had long been regarded as the ’fuel of future’, due to its high calorific value of combustion and low environmental pollution [1]. For instance, the vehicles powered by hydrogen cells release zero emissions of CO_2_ if the hydrogen fuel can be produced from renewable circulations [2,3]. However, in real practice, to fully utilize hydrogen as an ideal energy carrier is usually challenging, with the largest hurdle being associated with its delivery and storage operations [4,5]. Traditional technologies for hydrogen storage usually require compression under high pressures (around 700 bar), possibly giving rise to safety issues; therefore, the development of materials-based storage media remains to be an important goal. During the past decades, various novel materials have been characterized and evaluated by researchers for hydrogen storage, including liquid hydrocarbons [6], metal hydrides [7,8,9,10], boron-containing compounds [11,12,13,14,15,16], etc. An ideally designed material for hydrogen storage should be competitive in both adsorption strength and operational conveniences, instead of underscoring just one attribute over the other.

**Figure 1:**
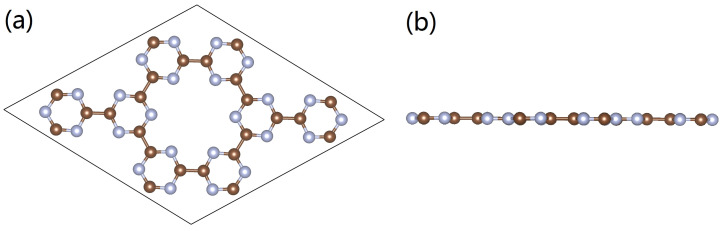
(**a**,**b**) Optimized configuration of the g-CN monolayer. Brown and silver spheres represent C and N atoms, respectively.

Over the past decades, two-dimensional (2D) graphene-like materials with porous structures were found to possess several superior properties for energy storage [17,18,19,20,21,22,23,24,25,26,27]. In one aspect, these kinds of graphene-like materials tend to possess large surface areas for efficient adsorptions, and some kinds of material storage capacities can even surpass 10 wt% [17,18,19,20,21,22,23,24,25,26,27,28,29,30]; in another aspect, their natural porosity enables them to easily accommodate active atoms or clusters to favorably change their electronic structures, and to further enhance their adsorption abilities [31,32,33,34,35,36,37,38,39,40]. In recent years, the successful synthesis of one promising graphene-like 2D material, carbon nitride (g-CN), was realized through the reaction between Na and C_3_N_3_Cl_3_, and under solvothermal conditions [41], the optimized configuration is presented in Figure 1. In each of its units, there exist six pyridinic N atoms, and the band gap is estimated to be 2.73 eV. With such a porosity, the g-CN can easily accommodate metal atoms for functional modification. Chen et al. reported that with the decoration of Li atoms, g-CN displays high performance in hydrogen storage [42]. It had been well recognized that the doped metal atoms can bind with pristine 2D materials, and that the binding structure can furthermore impact on its adsorption capabilities [35]; however, to fundamentally identify the correlation between the electronic structure of the metal-doped g-CN and its performance in gas adsorption, more studies are needed.

Metallic Al has three outer electrons, making it easy for Al to bind with atoms with a high electro-withdrawing power. Thus, in this study, we computationally solved the electronic structure of Al-doped g-CN by employing density functional theory (DFT) calculations. Furthermore, we also evaluated its potential in hydrogen storage and identified the factors that are crucial for its performance. We believe that the findings provided by this study will be valuable and will largely assist in the development of novel 2D materials with improved performance for renewable energy storage.

## 2. Materials and Methods

DFT calculations were conducted with the Vienna Ab-initio Simulation Package (VASP) [43,44]. The lattice parameters of the g-CN supercell were set to *a* = *b* = 14.24 Å. The generalized gradient approximation developed by Perdew–Burke–Ernzerhof [45,46] was applied for exchange-correlation energies calculations. A plane wave basis was employed to expand the electronic states, and the energy cutoff was set to 520 eV. The projector augmented wave method was applied to describe the core–valence interactions [47]. The Al-doped g-CN system contains 52 atoms; therefore, for a 2 × 2 supercell, gamma-centered k-point grid of 3 × 3 × 1 was applied for Brillouin zone sampling [48,49]. Van der Waals corrections were considered by implementing the DFT-D2 method [44]. A 30 Å vacuum layer was adopted to isolate the periodic slabs along the *c* direction. Structural relaxations were realized using the conjugate-gradient algorithm. The charge transfer among the doped Al atoms and the pristine g-CN surface were estimated using the Bader charge analysis [50]. The applied convergence criterion of the Hellmann–Feynman forces was set to 0.01 eV/Å, and $10^{-5}$ eV for the electronic structures calculations and geometry optimizations. All the computational parameters were set with a balance between cost and accuracy.

For the Al atoms on the pristine g-CN, the adsorption energy was obtained by the equation below:

(1) $$E_{ad}\left(\mathrm{Al}\right)=\left[E\left({\mathrm{Al}}_{k}\circ C_{24}N_{24}\right)-E\left(C_{24}N_{24}\right)-kE\left(\mathrm{Al}\right)\right]/k\left(\mathrm{Al}\right),$$

where *k*(Al) is the number of Al atoms, and *E* indicates an energy term. The adsorption energy per H_2_ molecule was calculated with the following equation:

(2) $$E_{ad}\left(H_{2}\right)=\left[E\left(nH_{2}•\mathrm{Al}\circ C_{24}N_{24}\right)-E\left(\mathrm{Al}\circ C_{24}N_{24}\right)-nE\left(H_{2}\right)\right]/n\left(H_{2}\right),$$

where *n*(H_2_) is the number of H_2_ molecules that are adsorbed by the Al-doped g-CN surface.

## 3. Results and Discussion

### 3.1. Structural Features of Al-Doped g-CN

**Figure 2:**
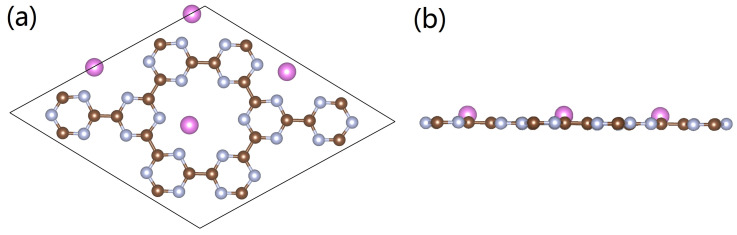
(**a**,**b**) Optimized configuration of the Al-doped g-CN monolayer. Brown, silver, and pink spheres represent C, N, and Al atoms, respectively.

To identify the possible adsorption sites for Al atoms on the surface of g-CN, sites close to the pyridinic N atoms either parallel or slightly above the plane were considered. After DFT optimizations, we found that the favorable adsorption site is located in the vicinity of two neighboring N atoms where the adsorbed Al atom is slightly off the plane. The corresponding adsorption energy per Al atom is −4.76 eV; and it tends to bind with the pyridinic N atoms. The specific configurations are presented in Figure 2. It is highly possible that such a binding between Al and N atoms is associated with charge transfer (more details will be discussed in the following section). In this study, we merely focused on the configuration that has one single Al atom at each pore of g-CN; the reason for this lies in the fact that due to steric hindrance, the accommodation of multiple metal atoms with larger sizes in the same pore may eliminate the adsorption capacity of the H_2_ molecules. However, for the doped atoms or clusters with a smaller size, such as Li, NLi_4_, etc., the placement of multiple units in one pore may enhance the adsorption performance.

**Figure 3:**
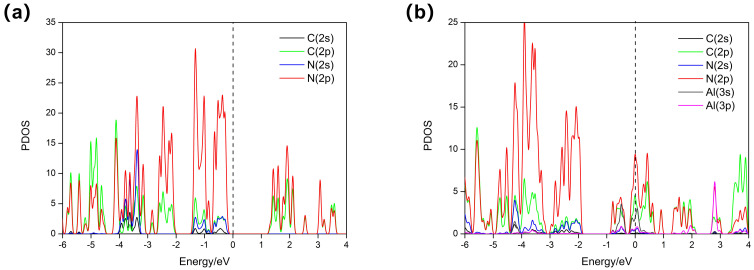
(**a**,**b**) The PDOS of the pristine g-CN and Al-doped g-CN for selected N, C, and Al atoms. The energy was plotted with respect to the Fermi energy.

**Figure 4:**
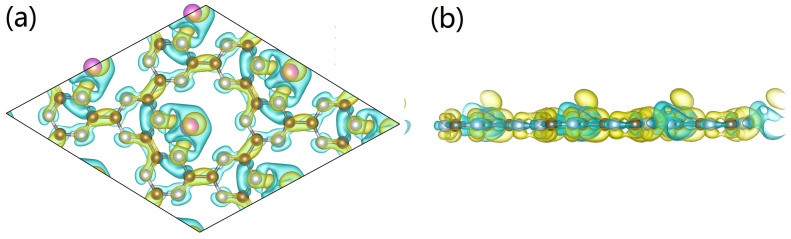
(**a**,**b**) Charge density difference for the Al-doped g-CN. Yellow regions indicate charge gain and the blue regions indicate charge loss. The isosurface of charge density is 0.0026 e/Bohr${}^{3}$.

From the calculated density of states (DOSs), we found the conductivity of Al-doped g-CN was enhanced, further confirming our presumption that there exist binding and charge transfers between the pyridinic N and adsorbed Al atoms. The partial density of states (PDOS) of pristine g-CN are presented for the 2p and 2s orbitals of C/N atoms in Figure 3a, and it is notable that these two orbitals are hybridized with each other. With the addition of Al atoms, we noticed that the mid-band states appear at the Fermi level, as can be seen in Figure 3b, and the overlap between the 3s/3p orbitals from the Al atoms and the hybrid orbitals of the C/N atoms further indicate the chemical binding and charge transfer between the Al atoms and g-CN. It is highly possible that the 3s/3p electrons of Al atoms are first hybridized, and then the Al atoms can bind with the two neighboring N atoms. Additionally, due to the fact that C and N are hybridized, the electrons from Al atoms can be transferred to the whole surface of g-CN; therefore, the electronic structure of g-CN may be largely changed. To clearly elucidate the chemical interactions between the Al atoms and g-CN, a Bader analysis was performed; the estimated amount of transferred charge from Al atoms is 1.2 e${}^{-}$. Such a value is smaller than that of Mg atoms [29], and we can further realize that the overall electronic structure of the metal-doped 2D materials is also associated with the electron-donating power of the deposited metal atoms. The charge density difference for Al-doped g-CN is presented in Figure 4, and we can see that the higher electronegativity of C/N makes them easily obtain electrons from the Al atoms. This is consistent with our discussion. Moreover, we also notice that the doped Al atom loses electrons and tends to be polarized, and a local electronic field with some regions displaying a higher electropositivity can be formed around it. In fact, the addition of Al atoms can successfully transform the semiconducting g-CN into a conducting one. We can also expect the Al-doped g-CN with a superior electronic structure can perfom well in H_2_ adsorptions.

**Figure 5:**
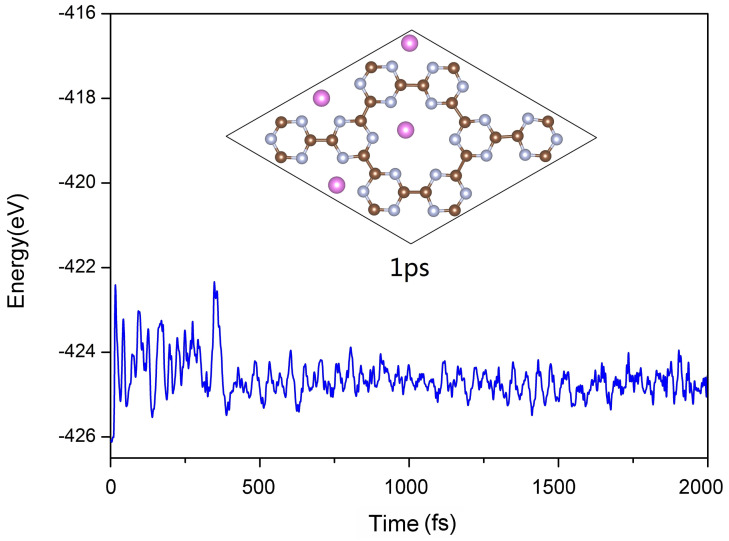
First−principles MD simulation of the Al-doped g-CN at a temperature of 300 K. The time step is set to 0.5 fs.

The thermodynamics stability of Al-doped g-CN was also evaluated in this study. First-principles molecular dynamics (MD) simulations of this composite material were performed under NVT ensemble; as can be seen from Figure 5, at a temperature of 300 K, its structure is proven to be stable, further demonstrating its applicability in real practice.

### 3.2. Hydrogen-Storage Performance Using Al-Doped g-CN

**Figure 6:**
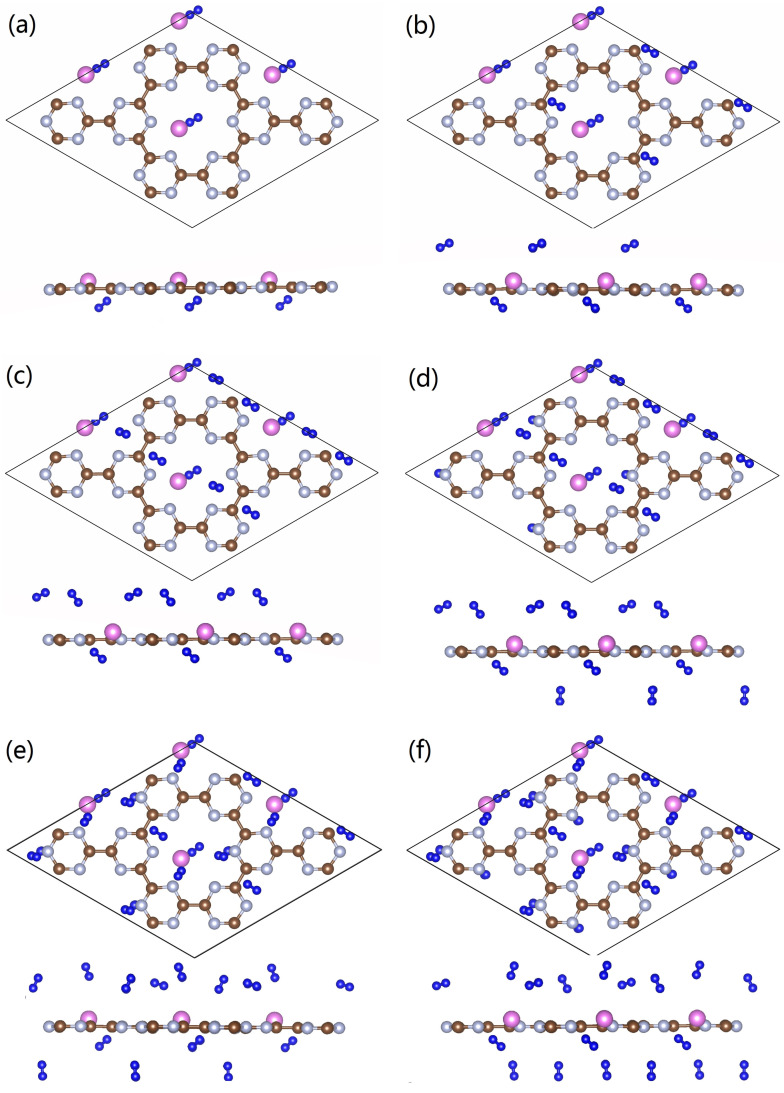
(**a**–**f**) The optimized configurations of the Al-doped g-CN with multiple adsorbed H_2_ molecules (4–24).

**Figure 7:**
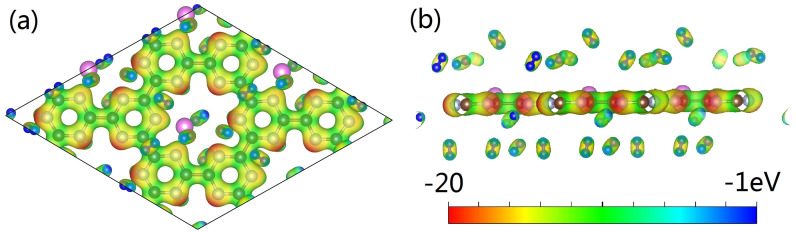
(**a**,**b**) The map of electrostatic potential distribution for the Al-doped g-CN with adsorbed H_2_ molecules.

After clarifying the structural features of the Al-doped g-CN, we continue to evaluate its performance in hydrogen storage. The optimized configurations of Al-doped g-CN with multiple adsorbed H_2_ molecules are presented in Figure 6. To detect suitable adsorption sites, we referred to the map of electronic potential distribution, as shown in Figure 7. It is observable that there are many active sites of adsorption, around the Al/N atoms. Thus, we placed H_2_ molecules around these Al/N atoms, and ab initio-based calculations were performed to locate the minimum of potential energy surface. As discussed in the previous section, each Al atom had transferred its partial charges to the surface of g-CN, and displayed an electropositive charge. Then, the local electronic field around the Al atoms can further induce the polarization of nearby H_2_ molecules, enhancing its adsorption ability. From the map of charge density difference for Al-doped g-CN with adsorbed H_2_, as presented in Figure 6, we noticed that within each polarized H_2_ molecule, one H loses electrons and shows electronegativity, while the other gains electrons and displays electropositivity; such a phenomenon is also observable for other metal-decorated 2D materials. Thus, we can conclude that the electrostatic interaction among Al atoms and these polarized H_2_ molecules can be attributed to a dipole-charged one. The highest adsorption energy of a single H_2_ molecule by an Al atom can reach up to −0.309 eV.

**Figure 8:**
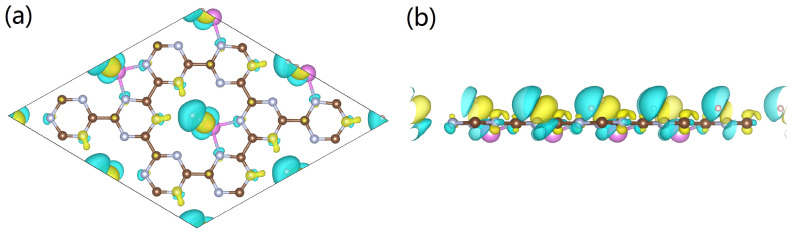
(**a**,**b**) Charge density difference for the Al-doped g-CN with multiple adsorbed H_2_ molecules. Yellow regions indicate charge gain and blue regions indicate charge loss. The isosurface of charge density is set to 0.0016 e/Bohr${}^{3}$.

To further estimate the highest adsorption capacity of H_2_, many possible sites were considered, and we found that each 2 × 2 supercell containing four Al atoms can accommodate, at most, 36 H_2_ molecules. Besides the region surrounding the Al atoms, H_2_ molecules can also be adsorbed by C/N atoms. For these adsorbed H_2_ molecules in the vicinity of non-metallic sites, they tend to be parallel with the plane. Such a binding geometry may be largely caused by polarization effects, as the middle region of H−H bonds tend to show electropositivity, and this region can interact with the C/N atoms that are highly electronegative to form a hydrogen bonding-like interaction (more details can be found in Figure 7 and Figure 8). The H_2_ molecules distributed around the Al atoms tend to be bound vertically to the plane, similar to the case of Mg doping. The adsorption energies of H_2_, the lengths of the H−H bonds, and the adsorption capacities of each configuration are presented in Table 1; it is observable that all H−H bonds in optimized configurations are slightly stretched. It is notable that the averaged adsorption energy per H_2_ decreases with the total adsorption capacity, indicating that for each active site, due to steric hindrance, there exists a maximum number of adsorbed H_2_ molecules.

## 4. Conclusions

The electronic structure of Al-doped g-CN was systematically solved via ab initio calculations, and its potential for hydrogen storage was evaluated. With the addition of Al atoms, the highest hydrogen-storage capacity of g-CN can reach up to 6.15 wt%. The Al atoms can bind with N atoms, and there exists a partial charge transfer between these Al atoms and the pristine g-CN. The adsorption energy of each Al atom in the pore of g-CN is around −4.67 eV. The higher electropositivity of the Al atoms can induce the polarization of the adsorbed H_2_ molecules, further enhancing the electrostatic interactions among them. The electronic potential distribution map of Al-doped g-CN was also presented in this study, and the most active sites for adsorption, along with the corresponding adsorption energies, were described. With this fundamental investigation, the adsorption mechanism of graphene-like 2D materials was clearly elucidated; and the most important factors associated with their adsorption performance were summarized. We believe that the findings provided by this study will largely facilitate the development of advanced nanostructure-based materials.

## Figures and Tables

**Table 1:** The adsorption energy (E${}_{Ad}$, in eV) per H_2_ on Al-doped g-CN, the averaged H−H bonding length (in Å), and the hydrogen-storage capacity (wt%).

System	Adsorption E	H−H Bond	Capacity
Al_4_C_24_N_24_ + 4H_2_	−0.31	0.787	1.10
Al_4_C_24_N_24_ + 8H_2_	−0.19	0.770	2.14
Al_4_C_24_N_24_ + 12H_2_	−0.15	0.765	3.17
Al_4_C_24_N_24_ + 16H_2_	−0.13	0.762	4.12
Al_4_C_24_N_24_ + 20H_2_	−0.13	0.762	5.18
Al_4_C_24_N_24_ + 24H_2_	−0.11	0.759	6.15

## Data Availability

The data that support the findings of this study are available from the corresponding author, upon reasonable request.
